# Contrast enhanced ultrasound and laser doppler flowmetry capture distinct aspects of tissue microvascular perfusion in nonhuman primates

**DOI:** 10.14814/phy2.71038

**Published:** 2026-08-07

**Authors:** Emily Attrill, Shannon Krainiak, McKinley Santiago, Kylie Kavanagh

**Affiliations:** ^1^ Department of Pathology Wake Forest University School of Medicine (WFUSM) Winston‐Salem North Carolina USA

**Keywords:** adipose tissue, contrast enhanced ultrasound, laser doppler flowmetry, microvascular perfusion, skeletal muscle, type 2 diabetes

## Abstract

Microvascular perfusion in skeletal muscle (SkM) and subcutaneous adipose tissue (AT) is critical for insulin‐mediated glucose uptake and is impaired in type 2 diabetes (T2D). Quantifying perfusion using contrast enhanced ultrasound (CEU) is technically challenging, prompting a need for simpler methods such as laser Doppler flowmetry (LDF). This study evaluated the relationship between LDF‐derived and CEU‐derived perfusion measures in SkM and subcutaneous AT of control and T2D nonhuman primates. Fourteen *cynomolgus macaques* (control *n* = 6; T2D = 8) underwent simultaneous CEU and LDF measurements at baseline and post‐intravenous dextrose (300 mg/kg). CEU‐derived perfusion and LDF‐derived perfusion were compared across conditions in SkM and subcutaneous AT. In SkM, CEU‐derived perfusion doubled post‐dextrose in controls whereas this response was attenuated in T2D animals. In subcutaneous AT, CEU‐derived perfusion increased post‐dextrose in T2D animals. LDF did not detect significant post‐dextrose changes in either tissue. There was no correlation between absolute CEU‐ and LDF‐derived perfusion at baseline; however, the percent change in SkM perfusion was positively correlated between methods. These findings suggest that LDF should not be considered interchangeable with CEU for quantifying tissue microvascular perfusion but may provide complementary information about relative changes in SkM perfusion when an intervention is applied to alter hemodynamics.

## 1 INTRODUCTION

In a healthy person, skeletal muscle (SkM) and adipose tissue (AT) combined are responsible for around 80% of insulin‐mediated glucose uptake following a meal (Defronzo et al., 1985; Defronzo, 2009). Under resting postprandial conditions, the rate‐limiting aspect of this process in both SkM and AT is the delivery of glucose to cells via the microvasculature (resistance arterioles and capillaries; (Barrett et al., 2011; Premilovac et al., 2013)), which is largely facilitated by insulin‐stimulated vasodilation. It is well established that disruption of insulin‐stimulated vasodilation in the microvasculature is one of the earliest hallmarks of metabolic disease. Impaired vasodilation precedes insulin resistance in the macrovasculature and at the myocyte and adipocyte (Hirano, 2018; Konishi et al., 2017; Kubota et al., 2011; St‐Pierre et al., 2010), which contributes to metabolic pathologies such as hyperinsulinemia, hypertriglyceridemia, and hyperglycemia. This strongly implicates the microvasculature, and subsequent microvascular dysfunction, in the development and progression of metabolic diseases such as type 2 diabetes (T2D). Despite the recognized importance of microvascular perfusion in metabolic disease, accurately quantifying tissue‐level microvascular perfusion remains technically challenging.

Whilst large fluctuations in microvascular perfusion occur in SkM and AT in response to certain stimuli (insulin (Barrett et al., 2011; Virtanen et al., 2002), exercise (Parker et al., 2021; Simonsen et al., 2004)), there are also more nuanced, moment‐to‐moment alterations in microvascular tone (Blackwood et al., 2017; Schmidt et al., 1993; Sotornik et al., 2012) that have dramatic effects on basal capillary perfusion and therefore the potential for nutrient exchange. Therefore, techniques capable of capturing both rapid temporal fluctuations and gross changes in tissue perfusion are critical for capturing the complex relationship between nutrient uptake and tissue perfusion in healthy and metabolically unhealthy tissue. Whilst multiple diagnostic techniques exist to detect blood flow changes in situ, including magnetic resonance imaging (Damon et al., 2007; Zhang et al., 2015), computed tomography perfusion (Chou et al., 2020; Liu et al., 2023), and positron emission topography (Honka et al., 2018), the applicability of these techniques to the field of microvascular research is poor due to limited spatial resolution, low sampling frequency, and accessibility (equipment and personnel costs to collect and analyze images). These limitations highlight the need for techniques that balance spatial resolution, temporal sampling, and accessibility.

Contrast enhanced ultrasound (CEU) is a safe, non‐invasive imaging modality that utilizes an intravenous contrast agent (<5 μm diameter, typically phospholipid or protein microbubbles filled with inert gas) to visualize and measure microvascular perfusion in real‐time. Whilst CEU has been used extensively to assess SkM and subcutaneous AT microvascular perfusion in both metabolically healthy and unhealthy humans (Coggins et al., 2001; Keske et al., 2009; Meijer et al., 2015; Roberts‐Thomson et al., 2022; Tobin et al., 2010) and animal models (Belcik et al., 2015; Chadderdon et al., 2016; Vincent et al., 2002), the high cost of equipment (ultrasound and contrast agent) and technical expertise required to collect and analyze the images limit the broad application of this research technique. Therefore, the aim of this study was to evaluate the relationship between Laser Doppler flowmetry (LDF) derived and CEU‐derived microvascular perfusion measures in SkM and subcutaneous AT of metabolically healthy and T2D nonhuman primates (NHPs).

LDF is a minimally invasive technique that provides continuous, real‐time assessment of microvascular perfusion within a small, localized tissue volume (Clough et al., 2009; Micheels et al., 1984). In contrast to CEU, LDF requires only an initial device investment with reusable probes that permit rapid and concurrent sampling of multiple regions with minimal technical skill required. Since LDF samples only a very small, localized tissue volume, it remains unclear whether these measurements reliably represent perfusion across an entire tissue. The OxyFlo™ LDF system has been applied across multiple organ systems in rodent, porcine, and human subjects (Entezari et al., 2025; Hsu et al., 2023; Loai & Cheng, 2025; Ospina‐Tascón et al., 2023), including SkM and AT where it was utilized to validate other techniques for measuring perfusion (MRI (Ganesh et al., 2019), ^133^Xe washout (Wellhöner et al., 2006)), and gather data in response to physiological challenges such as a breath hold (Wellhöner et al., 2006) or administration of vasoactive substances such as angiotensin II (Kuznetsova et al., 1998). Despite this, the application of LDF to preclinical research studies is limited. A recent study utilizing the OxyFlo™ Pro LDF to assess placental perfusion in pregnant African green monkeys (*Chlorocebus aethipops sabaeus*) found little correlation between LDF and CEU (Gonzalez‐Rodriguez et al., 2026). This group attributed this disparity to heterogeneous placental perfusion, variation in LDF probe placement across animals and timepoints, and a lack of intervention to manipulate placental perfusion during continuous LDF measures (Gonzalez‐Rodriguez et al., 2026). Extending this comparison to SkM and subcutaneous AT in NHPs is valuable as NHPs develop obesity‐associated insulin resistance and T2D with metabolic and vascular features that are more comparable to human disease than many rodent models. In addition, the invasive nature of LDF needle probes used to sample SkM and subcutaneous AT would be difficult to apply in human studies making NHPs a valuable translational model for this work. Therefore, we designed this study to evaluate the relationship between LDF‐derived and CEU‐derived measurements of microvascular perfusion in SkM and subcutaneous AT using healthy and T2D NHPs. We hypothesized that LDF‐derived perfusion would be positively associated with CEU‐derived perfusion across SkM and subcutaneous AT, particularly when expressed as within‐animal relative change following dextrose administration.

## 2 METHODS

### 2.1 Non‐human primate cohort

A total of 14 male and female cynomolgus macaques (*Macaca fascicularis*) were included in this study. Animals were middle‐aged to older and ranged from lean to obese (obesity defined by waist circumference >40 cm and percent body fat >30%). Animals were allocated to the control group (*n* = 6) or classified as T2D (*n* = 8) in accordance with the American Diabetes Association definition of two or more fasting blood glucose measures ≥126 mg/dL and glycosylated hemoglobin A1c (HbA1c) value >6.5% (see Table 1). Insulin therapy was prescribed in diabetic animals that were severely hypertriglyceridemic and experiencing weight loss. All animals were housed indoors, with the majority socially housed (paired housing with continuous full contact; two males singly housed). All animals were provided ad libitum access to food (standard old world NHP laboratory chow; Laboratory Diets 5038; LabDiet, St. Louis, MO) and water. Additionally, animals received supplementation with fresh fruits and vegetables and other enrichment foods such as popcorn. All animal procedures were performed according to the National Institute of Health Guide for Care and Use of Laboratory Animals. The study protocol was assessed and approved by the Wake Forest University School of Institutional Animal Care and Use Committee (protocol #A24‐008).

**TABLE 1:** Descriptive data of nonhuman primates (NHPs).

	Control (*n* = 6)	T2D (*n* = 8)	*p*‐value
Age (years)	12.8 ± 3.99	18.8 ± 2.18	**0.003**
Waist circumference (cm)	48.1 ± 8.18	51.4 ± 7.30	0.434
Weight (kg)	7.49 ± 2.50	9.71 ± 3.11	0.177
DEXA % body fat	43.3 ± 9.96	35.6 ± 7.99	0.153
Heart rate (beat/min)	145.9 ± 17.0	147.2 ± 18.6	0.900
Systolic blood pressure (mmHg)	91.8 ± 36.8	106.0 ± 28.8	0.434
Diastolic blood pressure (mmHg)	40.4 ± 18.0	57.9 ± 12.6	0.054
Mean arterial pressure (mmHg)	57.6 ± 23.3	73.9 ± 17.2	0.155
Fasting blood glucose (mg/dL)	65.7 ± 24.0	255.9 ± 76.4	**<0.001**
Fasting plasma insulin (mU/L)	33.5 ± 18.79	33.3 ± 29.31	0.991
Fasting HOMA‐IR	26.9 ± 36.2	1667 ± 1096.4	**0.003**
Glycosylated A1c (%)	4.25 ± 0.29	8.78 ± 1.58	**<0.001**

*Note*: Bold *p*‐values indicate statistical significance (*p* < 0.05).

Blood glucose and insulin concentration, and homeostatic model assessment (HOMA‐IR) were each calculated as previously described (Kavanagh et al., 2017). All male animals were classified as diabetic. Animals in the control and T2D group had similar body composition and broad hemodynamic variables (see Table 1).

### 2.2 Study design

**FIGURE 1:**
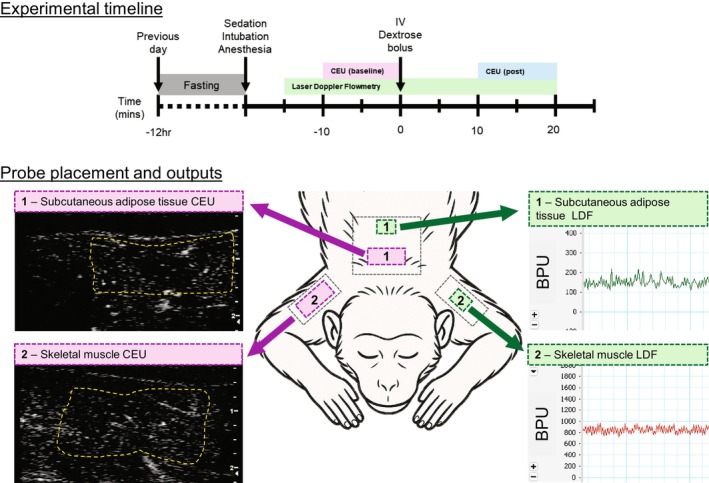
Experimental timeline and schematic of ultrasound and laser doppler probe placement and outputs Animals were fasted 12 h prior to procedure. Following sedation, intubation and anesthesia, Laser Doppler flow (LDF) probes (shown in green) were placed simultaneously in subcutaneous adipose tissue (AT) and skeletal muscle (SkM) to continuously record blood perfusion units (BPU) throughout. Contrast enhanced ultrasound (CEU) imaging was performed in subcutaneous AT and SkM sequentially at baseline (shown in pink) and 10 min post‐dextrose bolus (300 mg/kg; shown in blue). Post hoc CEU analysis involved selecting regions of interest to calculate microvascular perfusion, microvascular volume and microvascular flow velocity in subcutaneous AT and SkM.

This study involved collecting CEU and LDF measures in SkM and subcutaneous AT at baseline and following an intravenous dextrose bolus (300 mg/kg) to stimulate endogenous insulin secretion and induce insulin‐mediated hyperemia (depicted in Figure 1). For this procedure, animals were fasted for 12 h with continued access to water. If insulin therapy was prescribed, only regular short‐acting insulin was administered 16 h prior to measurements so that no exogenous insulin was in circulation while perfusion was assessed. Animals were sedated with intramuscular ketamine (15 mg/kg) prior to intubation and the placement of two IV catheters (20–22 gauge); one to continuously infuse propofol (0.3 mg/kg/min) for induction and maintenance on anesthesia and another to infuse contrast agent. Heart rate, blood pressure, respiratory rate, perfusion and vital signs were monitored every 10 min. Animals were positioned in ventral recumbency and the skin overlying the right and left triceps brachii, the right biceps femoris and the cervical and thoracic region of the dorsum were shaved and aseptically prepared. Monkeys received maropitant (1 mg/kg) prior to conclusion of procedure to prevent nausea on recovery.

#### 2.2.1 Contrast enhanced ultrasound (CEU)

We performed CEU imaging on SkM and subcutaneous AT using the LOGIQ S8 ultrasound system (GE HealthCare, USA) and a 5–12 MHz linear array probe (11L; GE HealthCare, USA). CEU imaging requires intravenous infusion of a contrast agent for microvascular imaging. Commercially available DEFINITY® microbubble contrast agent (Lantheus Medical Imaging) was used in this study. This agent was ‘activated’ by 45 s of vigorous shaking in a Vialmix® immediately prior to infusion as a 4.25% solution (diluted in 0.9% saline) at a rate of 2 mL/min. Using contrast mode on the ultrasound system, we visualized microbubble signal intensity at baseline and 10 min post‐dextrose bolus (see Figure 1) using the following imaging parameters: frame rate (22 Hz), mechanical index (0.15), gain (41 dB), dynamic range (66 dB), imaging depth (3 cm), and transducer focal point (2.4 cm). After a 2‐min steady‐state infusion, we captured a series of 30 s flash‐replenishment sequences. Briefly, this involves administration of a short, high‐energy pulse of ultrasound (mechanical index: 1.3) which destroys microbubbles within the imaging region. The return of microbubbles to the region is recorded in real time and is indicative of perfusion. A total of 14 replenishment sequences were captured per animal across the two timepoints. At baseline, 4 replicate replenishment sequences of subcutaneous AT were captured with the transducer perpendicular to the spine (approximately at the intersection between the cervical and thoracic regions). We then performed a series of 3 replicate replenishment sequences to capture baseline perfusion in the triceps brachii muscle. This protocol was then repeated 10 min following a 300 mg/kg dextrose bolus to induce microvascular vasodilation. Infusion of the contrast agent was paused between baseline and post bolus measures.

CEU images were exported as video files for offline processing and analysis using the Narnar application (narnar, LLC, USA). Regions of interest were placed over the SkM and subcutaneous AT (see Figure 1), and average microbubble signal (acoustic intensity; AI) across time was extracted for each replenishment sequence. All images were background subtracted to eliminate tissue signal and microbubble signal from large vessels (arteries and arterioles). The resulting signal therefore represents microvascular perfusion (Keske et al., 2009). Background subtracted acoustic intensity versus time was fitted to the function: *y = A* (1 – e^*−*β*(t)*^), where: *y* is acoustic intensity at time *t*, *A* is plateau acoustic intensity (microvascular blood volume; AI), and *β* is the rate constant (microvascular flow velocity; sec^−1^), as previously described (Coggins et al., 2001). Microvascular perfusion was determined by *A* × *β* (AI/s). All imaging and image analysis was performed by a single technician (EA) who was blinded to the animal's health status. Although the technician was not blinded to the dextrose status (baseline or post‐dextrose CEU measures), identical regions of interest based off baseline imaging were applied to all images, limiting the impact of bias in this study.

#### 2.2.2 Laser Doppler flowmetry (LDF)

We performed LDF (OxyFlo™ Pro; Oxford Optronix, UK) in subcutaneous AT (dorsal region over the mid‐thoracic vertebra; see Figure 1) and the SkM (left triceps brachii or right biceps femoris; see Figure 1). To insert probes, we first created a pathway into each tissue using a 14 gauge needle. Probes were positioned approximately 2 cm deep in subcutaneous AT and in the SkM to correspond with the depth of target tissues as measured by CEU. One flow probe was inserted into SkM and a second flow probe was inserted into subcutaneous AT to simultaneously measure flux in each tissue type for the duration of the procedure. Real‐time relative perfusion (arbitrary blood perfusion units; BPU) was recorded continuously in both tissues for the duration of the experiment using the integrated LabChart8 software. We identified a 1 min period of stable signal that aligned with baseline and post‐dextrose CEU imaging in both SkM and subcutaneous AT and extracted the median BPU for that time period. Median values were used to minimize the influence of transient fluctuations inherent to LDF signals.

### 2.3 Statistical analysis

This was an exploratory methodological study using an opportunistic cohort of clinically characterized NHPs. Therefore, a pre‐study power calculation was not performed. Sample size was determined by the availability of animals with paired CEU and LDF measurements across baseline and post‐dextrose conditions. The primary analysis focused on the relationship between CEU‐derived perfusion and LDF‐derived perfusion measures at each timepoint. Statistical analyses were performed using GraphPad Prism (GraphPad Software, USA; v10.5.0) and Statistica software (TIBCO Software Inc., USA; v14.2.0.18). Descriptive data are presented as mean ± standard deviation and all other data are presented as mean ± standard error of the mean. Replicate CEU replenishment sequences were averaged to generate a single data value per animal for each tissue at either timepoint. Normality of all variables was assessed, and variables were log transformed where appropriate prior to statistical testing. Differences between groups and the effect of dextrose administration on tissue perfusion were evaluated using ANCOVA with age and baseline value included as covariates. Age was included as a variable known to independently modify microvascular function and was different between control and T2D groups (see Table 1). Pearson correlation analyses were performed to assess relationships between CEU‐derived perfusion measures and LDF‐derived perfusion at each timepoint and when expressed as percent change from baseline. As an additional baseline‐adjusted analysis, partial correlations were performed between post‐dextrose CEU‐ and LDF‐derived perfusion while controlling for baseline CEU‐derived perfusion. Statistical significance was defined as *p* < 0.05. As the enrollment of T2D NHPs was opportunistic based on availability at our NHP center, all male animals were classified as diabetic, and therefore sex could not be evaluated as an independent variable.

## 3 RESULTS

### 3.1 Animal response to dextrose bolus

**FIGURE 2:**
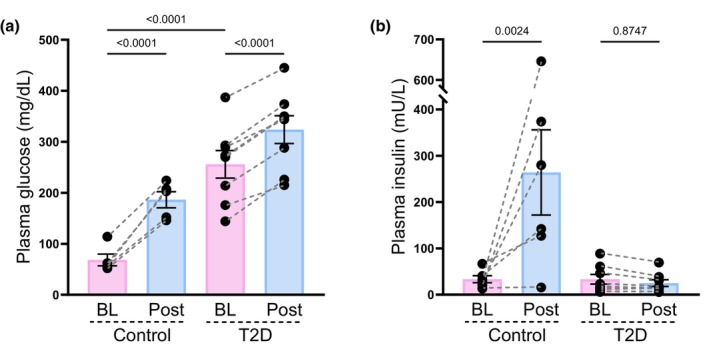
Dextrose administration increases plasma insulin in control animals but not T2D animals. (a) Plasma glucose and (b) plasma insulin concentration were measured at baseline (BL) and 10 min post‐dextrose bolus (Post) in control and T2D animals. Data is shown as mean ± SE.

Plasma glucose and insulin concentration were measured immediately prior, and 10 min following a dextrose bolus (see Figure 2). At baseline, plasma glucose concentration was elevated in T2D animals compared to controls (65.7 ± 9.79 vs. 255.9 ± 27.0 mg/dL, *p* < 0.001). Following dextrose administration, plasma glucose concentration increased in both control (65.7 ± 9.79 vs. 186.4 ± 15.7 mg/dL, p < 0.001) and T2D (255.9 ± 27.0 vs. 323.9 ± 27.3 mg/dL, p < 0.001) animals compared to baseline measures. At baseline, there was no difference in plasma insulin concentration between control and T2D animals (33.5 ± 7.67 vs. 33.3 ± 10.4 mU/L, *p* = 0.991). However, following dextrose administration, plasma insulin concentration increased 9‐fold in control animals (33.5 ± 7.67 vs. 264.1 ± 91.9 mU/L, *p* = 0.002) but was completely attenuated in T2D animals (33.3 ± 10.4 vs. 24.9 ± 7.38 mU/L, *p* = 0.875), which is consistent with impaired pancreatic insulin secretion characteristic of advanced T2D.

### 3.2 Skeletal muscle perfusion

**FIGURE 3:**
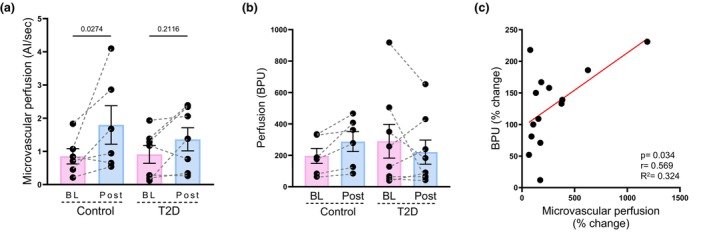
Percent change in SkM perfusion measured by CEU and LDF was correlated, although LDF‐derived perfusion did not independently detect insulin‐stimulated SkM hyperemia in control animals. SkM perfusion was measured by (a) CEU and (b) LDF at baseline (BL) and post‐dextrose administration in (Post) control and T2D animals. (c) Time matched CEU and LDF data values were converted to percent change from baseline to undergo Pearson correlation analysis (results shown). Data is shown as mean ± SE (A and B) or percent change from baseline (C).

Microvascular perfusion in SkM was quantified at baseline and following a dextrose bolus using CEU (see Figure 3a). Baseline SkM microvascular perfusion did not differ between control and T2D animals (0.85 ± 0.23 vs. 0.80 ± 0.26 AI/s, *p* = 0.457) or following a dextrose bolus (1.79 ± 0.58 vs. 1.36 ± 0.35 AI/s, *p* = 0.508). However, within individual changes following dextrose administration indicated that SkM perfusion doubled in control animals (0.85 ± 0.23 vs. 1.79 ± 0.58 AI/s, *p* = 0.027), an effect that was attenuated in T2D animals (0.91 ± 0.27 vs. 1.36 ± 0.35 AI/s, *p* = 0.212). CEU‐derived perfusion was also separated into its individual components: microvascular blood volume and microvascular flow velocity. In SkM, neither microvascular blood volume nor microvascular flow velocity differed between groups at baseline or following dextrose administration in the control or T2D groups (see Figure S1).

In addition to CEU, SkM perfusion was simultaneously measured using LDF (see Figure 3b). SkM perfusion measured by LDF did not differ between control and T2D animals at baseline (196.1 ± 47.7 vs. 289.7 ± 107.0 BPU, *p* = 0.775) or following dextrose administration (287.4 ± 63.1 vs. 220.6 ± 76.8 BPU, *p* = 0.958). In contrast to CEU, LDF did not detect a significant change in SkM perfusion following dextrose administration in the control group (196.1 ± 47.7 vs. 287.4 ± 63.1 BPU, *p* = 0.205) or the T2D group (289.7 ± 107.0 vs. 220.6 ± 76.8 BPU, *p* = 0.264).

To assess the relationship between CEU and LDF, we performed correlation analyses independent of health status. Absolute SkM microvascular perfusion measured by CEU did not correlate with LDF‐derived perfusion at baseline (*r* = 0.072, *R* ^2^ = 0.005, *p* = 0.806; see Figure S2) or following dextrose (*r* = 0.121, *R* ^2^ = 0.015, *p* = 0.681; see Figure S2). The percentage change in CEU‐derived SkM perfusion following dextrose administration positively correlated with percentage change measured by LDF (*r* = 0.5688, *R* ^2^ = 0.324, *p* = 0.034; see Figure 3c). However, this association was not retained in a partial correlation between CEU‐ and LDF‐derived perfusion after controlling for baseline CEU perfusion (*r* = 0.183, *R* ^2^ = 0.033, *p* = 0.550). There was no correlation between LDF‐derived SkM perfusion and the individual CEU‐derived components of microvascular perfusion, including microvascular blood volume or microvascular flow velocity at baseline, post‐dextrose, or when expressed as percent change from baseline (see Figure S2).

### 3.3 Subcutaneous adipose tissue perfusion

**FIGURE 4:**
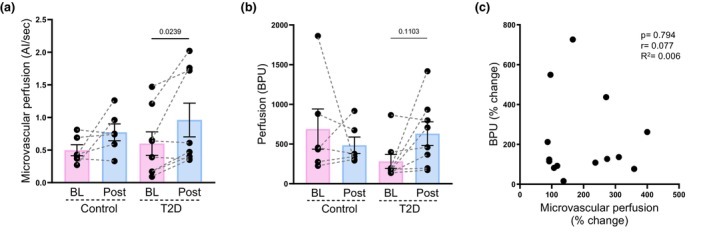
There was no correlation between CEU‐ and LDF‐derived perfusion measures in subcutaneous AT. Subcutaneous AT perfusion was measured by (a) CEU and (b) LDF at baseline (BL) and post‐dextrose administration (Post) in control and T2D animals. (c) Time matched CEU and LDF data values were converted to percent change from baseline to undergo Pearson correlation analysis (results shown). Data is shown as mean ± SE (A and B) or percent change from baseline (C).

Microvascular perfusion in subcutaneous AT was also quantified at baseline and following a dextrose bolus using CEU (see Figure 4a). There was no difference in subcutaneous AT perfusion between control and T2D animals at baseline (0.50 ± 0.09 vs. 0.60 ± 0.18 AI/s, *p* = 0.347) or following a dextrose bolus (0.77 ± 0.13 vs. 0.96 ± 0.26 AI/s, *p* = 0.576). In contrast to SkM, dextrose administration did not alter subcutaneous AT perfusion in control animals (0.50 ± 0.09 vs. 0.77 ± 0.13 AI/s, *p* = 0.115) however, increased subcutaneous AT perfusion in T2D animals (0.60 ± 0.18 vs. 0.96 ± 0.26 AI/s, *p* = 0.024). CEU‐derived microvascular perfusion in subcutaneous AT was also separated into microvascular blood volume and microvascular flow velocity. Microvascular blood volume did not differ between groups at either time point in control or T2D animals. In contrast, subcutaneous AT microvascular flow velocity was elevated following dextrose administration in control animals (0.12 ± 0.02 vs. 0.19 ± 0.02 s^−1^, *p* = 0.043; see Figure S3). Whilst subcutaneous AT flow velocity also appeared to increase following the dextrose bolus in T2D animals (0.16 ± 0.01 vs. 0.21 ± 0.02 s^−1^; see Figure S3), this relationship did not reach statistical significance (*p* = 0.087).

In addition to CEU, subcutaneous AT perfusion was simultaneously measured using LDF (see Figure 4b). LDF‐derived subcutaneous AT perfusion did not differ between control and T2D groups at baseline (688.6 ± 253.0 vs. 280.6 ± 88.6 BPU, *p* = 0.113) or following dextrose administration (484.6 ± 103.9 vs. 631.0 ± 148.9 BPU, *p* = 0.307). In contrast to CEU, LDF did not detect a significant change in subcutaneous AT perfusion following dextrose in control animals (688.6 ± 253.0 vs. 484.6 ± 103.9 BPU, *p* = 0.402). Whilst not statistically significant, LDF‐derived subcutaneous AT perfusion appeared to increase following dextrose administration in T2D animals (280.6 ± 88.6 vs. 631.0 ± 148.9 BPU, *p* = 0.110), a trend consistent with CEU observations.

To assess the relationship between CEU and LDF, we performed correlation analyses across the full cohort independent of health status. Although absolute subcutaneous AT microvascular perfusion measured by CEU did not correlate with LDF‐derived perfusion at baseline (*r* = 0.208, *R* ^2^ = 0.043, *p* = 0.475; see Figure S2), there was a positive correlation between LDF‐derived perfusion and CEU‐derived perfusion post‐dextrose values (*r* = 0.5751, *R* ^2^ = 0.331, *p* = 0.031; see Figure S2). Percent change in CEU‐derived subcutaneous AT perfusion did not correlate with percent change measured by LDF (*r* = 0.077, *R* ^2^ = 0.006, *p* = 0.794; see Figure 4c). In contrast, a partial correlation between CEU‐ and LDF‐derived perfusion controlling for baseline CEU perfusion identified a significant positive association (*r* = 0.605, *R* ^2^ = 0.366, *p* = 0.022). There was no correlation between LDF‐derived subcutaneous AT perfusion and microvascular blood volume or microvascular flow velocity measured by CEU at baseline (see Figure S2) or when expressed as percent change from baseline. However, following dextrose, LDF‐derived subcutaneous AT perfusion was positively correlated with CEU‐derived microvascular blood volume (*r* = 0.645, *R* ^2^ = 0.416, *p* = 0.013; see Figure S2).

## 4 DISCUSSION

The aim of this study was to evaluate the relationship between LDF‐derived perfusion measurements and CEU‐derived estimates of microvascular perfusion in SkM and subcutaneous AT. CEU detected the expected increase in SkM perfusion following a dextrose‐induced rise in plasma insulin in metabolically healthy animals, a response which was attenuated in animals with T2D. In contrast, LDF did not detect significant changes in perfusion following dextrose administration in either SkM or subcutaneous AT. Despite this, the percent change in SkM perfusion measured by LDF correlated with the percent change measured by CEU across the cohort, suggesting that while LDF may not accurately quantify absolute microvascular perfusion, it may capture relative changes in perfusion under certain physiological conditions. These findings are consistent with methodological differences between CEU and LDF and suggest that the two techniques quantify distinct aspects of microvascular blood flow.

Quantification of microvascular perfusion by CEU requires intravenously administered phospholipid microbubbles filled with inert gas. Microbubbles are similar in size and rheology to erythrocytes (Hsu et al., 2011; Vincent et al., 2002) and remain in the vasculature (Lindner et al., 2002). When exposed to low energy (low mechanical index) ultrasound, microbubbles oscillate in size and generate a strong acoustic signal enabling high‐contrast, continuous imaging of the microvasculature (Lee et al., 2017; Sjøberg et al., 2011). Subsequent exposure to high energy (high mechanical index) ultrasound pulses destroys microbubbles, enabling quantification of microbubble (and hence blood) refill kinetics which reflect microvascular blood volume, microvascular flow velocity, and microvascular perfusion (Lee et al., 2017; Sjøberg et al., 2011). In contrast, LDF relies on the delivery of low‐power laser light via a fiber‐optic probe. Upon interacting with moving erythrocytes, photons undergo a Doppler frequency shift, whereas photons reflected by static structures, such as myocytes and adipocytes, do not (Clough et al., 2009; Micheels et al., 1984). The magnitude and distribution of this Doppler shift reflect both erythrocyte velocity and concentration, and approximate perfusion within a small tissue volume (Clough et al., 2009; Micheels et al., 1984).

In this study, CEU reproduced an expected physiological response to elevated plasma insulin concentration following dextrose administration. In metabolically healthy animals, SkM microvascular perfusion approximately doubled following the dextrose bolus, consistent with previous studies demonstrating insulin‐stimulated microvascular recruitment in SkM (Barrett et al., 2011; Coggins et al., 2001; Vincent et al., 2002). It is well established that the microvascular effect of insulin occurs through activation of the IRS‐1/PI3K/AkT/eNOS pathway in endothelial cells (Kubota et al., 2011; Montagnani et al., 2002; Vincent et al., 2003). This pathway stimulates nitric oxide production which vasodilates resistance arterioles, increasing capillary perfusion and subsequently glucose delivery (Barrett et al., 2011; Coggins et al., 2001), a response that is blunted in T2D animals, consistent with microvascular insulin resistance, reduced activation of the IRS‐1/PI3K/AkT/eNOS, and limited capillary recruitment in SkM (Clerk et al., 2006; Keske et al., 2009; Kubota et al., 2011; Vincent et al., 2002). However, as dextrose stimulates endogenous insulin secretion rather than imposing matched hyperinsulinemia, the attenuated SkM perfusion response in T2D animals should be interpreted in the context of both impaired insulin secretion and vascular insulin resistance. Importantly, the ability of CEU to detect these expected physiological patterns supports its use for assessing microvascular perfusion responses in the present NHP cohort.

Despite the clear and physiologically sound changes in SkM perfusion detected by CEU, LDF did not detect significant changes in SkM perfusion following dextrose administration. This discrepancy likely reflects fundamental differences in how CEU and LDF measure blood flow. CEU quantifies microvascular perfusion by independently estimating microvascular blood volume and flow velocity based on microbubble refill kinetics across a defined imaging field (approximately 1100–1700 mm^3^; 11L linear probe and 3 cm depth). In contrast, LDF measures the Doppler frequency shift generated by moving erythrocytes within a much smaller tissue volume (approximately 1 mm^3^). Consequently, LDF provides a localized estimate of erythrocyte flux (erythrocyte velocity and concentration) in arbitrary units rather than an absolute physiological measure of perfusion across a tissue. Given these methodological differences, direct equivalence between CEU‐ and LDF‐derived perfusion measurements would not necessarily be expected.

The limited agreement between CEU and LDF‐derived perfusion measures in the present study challenges prior work demonstrating concordance between the techniques for detecting SkM flowmotion patterns in rats following pharmacological modulation of vascular tone (Blackwood et al., 2017). However, that study compared whether CEU and LDF detected similar flowmotion patterns in rat SkM, rather than whether the two methods produced comparable quantitative perfusion measures following a stimulus. In contrast, the present findings are more consistent with recent work in the NHP placenta, where CEU and LDF produced discordant estimates of placental perfusion, likely due to heterogeneous perfusion and variability in localized LDF probe sampling (Gonzalez‐Rodriguez et al., 2026). Together, these findings suggest that agreement between CEU and LDF depends on the study model, tissue examined, the outcome measured, the physiological stimulus, and the sampling volume of each technique (Clough et al., 2009). It is possible that in larger animal models such as NHPs, localized LDF measurements may be particularly sensitive to probe placement and less representative of perfusion across the broader CEU imaging region, especially in heterogeneous tissue such as the placenta and subcutaneous AT.

While absolute measurements of SkM perfusion did not correlate between CEU and LDF, the percent change in perfusion following dextrose administration was positively correlated between the two techniques. Although insulin‐stimulated vasodilation in SkM is dynamic, it is primarily regulated by terminal arterioles which supply large downstream capillary units (Clerk et al., 2006; Segal, 2005). As a result, insulin‐mediated changes in SkM perfusion may occur relatively uniformly across the tissue. This may explain why the small tissue volume sampled by LDF was sufficient to reflect broader relative changes in SkM perfusion, as indicated by the positive correlation in percent change between CEU‐ and LDF‐derived perfusion measures. However, LDF did not independently detect significant within‐group changes in SkM perfusion following dextrose administration. Additionally, it is important to note that the relationship between CEU‐ and LDF‐derived perfusion was not retained when adjusted for baseline CEU perfusion, suggesting that the correlation is sensitive to how baseline perfusion is accounted for. Therefore, while LDF may provide complementary information about relative changes in SkM perfusion under certain conditions, caution is warranted when interpreting LDF‐derived estimates of SkM perfusion as a stand‐alone measure.

In contrast to SkM, the relationship between CEU and LDF measurements in subcutaneous AT was weaker and less consistent. Although CEU detected increased subcutaneous AT perfusion following dextrose administration in T2D animals, LDF did not detect corresponding changes, and percent changes in perfusion were not correlated between the two techniques except when accounting for baseline CEU perfusion. The discrepancies between LDF and CEU correlation in SkM and subcutaneous AT may relate to the substantially smaller sampling volume of LDF, which may be further compounded by obesity. In the present study, both control and T2D animals were classified as confirmed obese (body fat percentage >30% (Garside et al., 2022)), a condition associated with adipocyte hypertrophy and reduced capillary density (Crewe et al., 2017; Pasarica et al., 2009). Consequently, the small tissue volume sampled by LDF may contain even fewer perfused capillaries in this obese model, increasing the likelihood that small, local fluctuations in erythrocyte flux may disproportionately influence the data. This suggests that localized LDF measurements do not consistently represent subcutaneous AT perfusion in this obese NHP cohort.

Together, these findings across SkM and subcutaneous AT emphasize that CEU and LDF each capture distinct physiological aspects of microvascular perfusion. CEU provides a spatially integrated measure of microvascular blood volume, flow velocity, and perfusion across a defined tissue region, making it well suited for quantifying microvascular recruitment and perfusion capacity (Coggins et al., 2001; Sjøberg et al., 2011). In contrast, LDF provides continuous, high temporal resolution measurements of erythrocyte flux within a highly localized sampling volume (Clough et al., 2009; Micheels et al., 1984). While this approach may be valuable for monitoring dynamic changes in perfusion or detecting rapid fluctuations in microvascular flow, it does not necessarily represent total tissue perfusion or allow reliable comparisons between subjects. As such, LDF should be considered a complementary rather than interchangeable technique when compared with CEU for assessing microvascular perfusion.

Beyond comparing CEU and LDF, further insight into regulation of subcutaneous AT microvascular perfusion can be gained from examining the various components of CEU‐derived perfusion. In subcutaneous AT, the dextrose stimulus appeared to increase microvascular flow velocity without a corresponding increase in microvascular blood volume implying that total blood flow through the existing capillary network increased without expansion of the perfused capillary bed (Coggins et al., 2001; Sjøberg et al., 2011). This pattern suggests that subcutaneous AT perfusion following dextrose administration may be driven primarily by alterations in local flow kinetics rather than overall capillary recruitment (Keske et al., 2009). This may reflect reduced vascular resistance in upstream arterioles leading to local alterations in velocity and more uniform capillary flow (Segal, 2005) rather than recruitment of additional capillaries. Alternatively, changes in downstream vascular resistance or systemic perfusion pressure may similarly increase the pressure gradient across the microvascular bed, accelerating erythrocyte transit through capillaries without altering microvascular volume within the imaging field. These findings highlight the importance of understanding perfusion dynamics, particularly as alterations in microvascular flow kinetics are likely key determinants of nutrient delivery and substrate exchange in subcutaneous AT.

While this study provides valuable insights into the relationship between CEU‐ and LDF‐derived measures of tissue perfusion, several limitations should be acknowledged. This was a methodological study that utilized a well characterized cohort of NHPs with paired CEU and LDF measurements, rather than a prospectively powered study. These findings should therefore be interpreted as an exploratory comparison of CEU‐ and LDF‐derived perfusion measures to inform future prospective studies. The NHP cohort also included important demographic differences that could influence perfusion responses. Control and T2D animals differed in age, and although age was included as a covariate in the statistical models, the small sample size limited our ability to fully separate age‐related effects. Biological sex was also confounded by disease status, as all male animals were classified as T2D. Therefore, we were unable to determine whether microvascular perfusion was influenced by age, biological sex, disease status, or interactions between these factors. The dextrose stimulus and anesthetic conditions should also be considered when interpreting the findings. Dextrose was used to stimulate endogenous insulin secretion rather than impose matched hyperinsulinemia across groups. As such, post‐dextrose perfusion reflects both pancreatic insulin secretion and downstream vascular responsiveness, rather than isolated vascular responses to equivalent exogenous insulin exposure. In addition, key sedatives and anesthetics, including ketamine and propofol, alter whole body hemodynamics (Reves et al., 2005). Whether these agents modify the microvascular effects of insulin is largely unknown and warrants further investigation. Finally, placement of LDF probes introduces potential variability related to the tissue volume and type sampled between subjects, particularly in subcutaneous AT where perfusion heterogeneity is high (Crewe et al., 2017; Pasarica et al., 2009). Although the LDF probes remained in place between baseline and post‐dextrose measures, this approach did not account for variability in the tissue volume sampled between animals. This further emphasizes the importance of carefully interpreting localized LDF measurements when assessing whole‐tissue microvascular perfusion.

## 5 CONCLUSION

In summary, this study suggests that CEU and LDF capture distinct aspects of microvascular blood flow in NHPs. CEU detected the expected physiological changes in SkM perfusion following dextrose stimulation, whereas LDF did not detect significant within‐group changes. Although changes in SkM perfusion measured by LDF correlated with CEU‐derived percent changes across the cohort, this relationship did not extend to subcutaneous AT where spatial heterogeneity likely limits the representativeness of localized LDF measurements. Collectively, these findings indicate that LDF should not be considered interchangeable with CEU for quantifying tissue microvascular perfusion but may provide complementary information about relative changes in SkM microvascular perfusion when an intervention is applied to alter hemodynamics.

## AUTHOR CONTRIBUTIONS

**Emily Attrill:** Conceptualization; data curation; formal analysis; investigation; methodology. **Shannon Krainiak:** Data curation; project administration. **McKinley Santiago:** Data curation; project administration. **Kylie Kavanagh:** Conceptualization; data curation; formal analysis; funding acquisition; supervision.

## FUNDING INFORMATION

Department of Defense W8XWH‐21‐1‐0565.

## CONFLICT OF INTEREST STATEMENT

The authors declare no known conflicting financial interests or relationships that could have appeared to influence the work reported in this paper.

## ETHICS STATEMENT

The authors confirm adherence to the journals' ethical policies as noted on the author guidelines page. All animal studies were performed at Wake Forest University School of Medicine (WFUSM) and complied with international, national, and institutional guidelines for humane animal treatment, including those set forth by US National Research Council “Guide for the Care and Use of Laboratory Animals” and the US Public Health Service “Policy on Humane Care and Use of Laboratory Animals” and “Guide for the Care and Use of Laboratory Animals.” All studies were conducted following approval of the WFUSM Institutional Animal Care and Use Committee (protocol #A24‐008).

## Supporting information


**Figure S1.** There was no correlation between CEU‐derived microvascular blood volume or microvascular flow velocity and LDF‐derived perfusion measures in SkM. SkM (a) microvascular blood volume and (b) microvascular flow velocity was measured by CEU at baseline (BL) and following dextrose administration (Post) in control and T2D animals. (c) Microvascular blood volume and (D) microvascular flow velocity were converted to percent change from baseline and correlated with time matched with LDF data values. Data is shown as mean ± SE (a, b) or percent change from baseline (c, d).
**Figure S2.** There was no correlation between CEU measures and LDF measures in SkM however, LDF perfusion positively correlated with CEU‐derived microvascular blood volume and microvascular perfusion post‐dextrose in subcutaneous AT. Raw CEU measures were compared to raw LDF perfusion in SkM at baseline (a–c) and following dextrose (d–f). Similarly, raw CEU measures were compared with raw LDF perfusion in subcutaneous AT at baseline (g–i) and following dextrose administration (j–l). Pearson correlation was performed for each comparison and is shown.
**Figure S3.** There was no correlation between CEU‐derived microvascular blood volume or microvascular flow velocity and LDF‐derived perfusion measures in subcutaneous AT. Subcutaneous AT (a) microvascular blood volume and (b) microvascular flow velocity was measured by CEU at baseline (BL) and following dextrose administration (Post) in control and T2D animals. (c) Microvascular blood volume and (d) microvascular flow velocity were converted to percent change from baseline and correlated with time matched with LDF data values. Data is shown as mean ± SE (a, b) or percent change from baseline (c, d).

## Data Availability

The datasets used and/or analyzed during the current study are available from the corresponding author on reasonable request.
