# Supplementary material for: Contrast enhanced ultrasound and laser doppler flowmetry capture distinct aspects of tissue microvascular perfusion in nonhuman primates
Source: Physiol Rep. 2026 Aug 7;14(15):e71038. doi: 10.14814/phy2.71038 (PMC13451646; doi:10.14814/phy2.71038)
Supplement: Supplementary file 1 [file PHY2-14-e71038-s001.docx]

**Supplementary information**

Contrast enhanced ultrasound and laser Doppler flowmetry capture distinct aspects of tissue microvascular perfusion in nonhuman primates.

**Running title:** Microvascular perfusion: CEU vs LDF

**Keywords:** microvascular perfusion; contrast enhanced ultrasound; laser Doppler flowmetry; skeletal muscle; adipose tissue; type 2 diabetes.

**Authors:**

Emily Attrill, *Department of Pathology, Wake Forest University School of Medicine (WFUSM), Winston-Salem, NC, USA*, emily.attrill@advocatehealth.org

Shannon Krainiak, *Department of Pathology, Wake Forest University School of Medicine (WFUSM), Winston-Salem, NC, USA*, shannon.scarberry@advocatehealth.org

McKinley Santiago, *Department of Pathology, Wake Forest University School of Medicine (WFUSM), Winston-Salem, NC, USA*, mcKinley.santiago@advocatehealth.org

Kylie Kavanagh, *Department of Pathology, Wake Forest University School of Medicine (WFUSM), Winston-Salem, NC, USA*, kylie.kavanagh@wfusm.edu

**Data Availability Statement:** The datasets used and/or analyzed during the current study are available from the corresponding author on reasonable request

**Funding Statement:** Department of Defense W8XWH-21-1-0565

**Conflict of Interest Disclosure:** The authors declare no known conflicting financial interests or relationships that could have appeared to influence the work reported in this paper.

**Ethics Approval Statement:** The authors confirm adherence to the journals ethical policies as noted on the author guidelines page. All animal studies were performed at Wake Forest University School of Medicine (WFUSM) and complied with international, national, and institutional guidelines for humane animal treatment, including those set forth by US National Research Council “Guide for the Care and Use of Laboratory Animals” and the US Public Health Service “Policy on Humane Care and Use of Laboratory Animals” and “Guide for the Care and Use of Laboratory Animals.” All studies were conducted following approval of the WFUSM Institutional Animal Care and Use Committee (protocol #A24-008).

**Corresponding Author:** Emily Attrill; emily.attrill@advocatehealth.org; (ORCID id: 0000-0002-2047-0590)

**Supplementary figure 1. There was no correlation between CEU-derived microvascular blood volume or microvascular flow velocity and LDF-derived perfusion measures in SkM.** SkM (A) microvascular blood volume and (B) microvascular flow velocity was measured by CEU at baseline (BL) and following dextrose administration (Post) in control and T2D animals. (C) Microvascular blood volume and (D) microvascular flow velocity were converted to percent change from baseline and correlated with time matched with LDF data values. Data is shown as mean ± SE (A and B) or percent change from baseline (C and D).

**Supplementary figure 2. There was no correlation between CEU measures and LDF measures in SkM however, LDF perfusion positively correlated with CEU-derived microvascular blood volume and microvascular perfusion post-dextrose in subcutaneous AT.** Raw CEU measures were compared to raw LDF perfusion in SkM at baseline (A-C) and following dextrose (D-F). Similarly, raw CEU measures were compared with raw LDF perfusion in subcutaneous AT at baseline (G-I) and following dextrose administration (J-L). Pearson correlation was performed for each comparison and is shown.

**Supplementary figure 3. There was no correlation between CEU-derived microvascular blood volume or microvascular flow velocity and LDF-derived perfusion measures in subcutaneous AT.** Subcutaneous AT (A) microvascular blood volume and (B) microvascular flow velocity was measured by CEU at baseline (BL) and following dextrose administration (Post) in control and T2D animals. (C) Microvascular blood volume and (D) microvascular flow velocity were converted to percent change from baseline and correlated with time matched with LDF data values. Data is shown as mean ± SE (A and B) or percent change from baseline (C and D).
